# Syndrome canalaire du pied révélant un schwannome du nerf fibulaire superficiel: résultat d’un traitement chirurgical conservateur

**DOI:** 10.11604/pamj.2017.28.161.12439

**Published:** 2017-10-19

**Authors:** Mustafa Nkaoui, Youness Sasbou

**Affiliations:** 1 Service de Chirurgie Orthopédique et de Traumatologie, CHU Ibn Sina, Université Mohammed V, Souissi, Rabat, Maroc

**Keywords:** Schwannome, nerf fibulaire superficiel, syndrome canalaire, Schwannoma, superficial peroneal nerve, entrapment neuropathy

## Abstract

We report the case of a 61-year old patient with no particular previous history who complained of pain with paraesthesias at the level of the anterolateral compartment of the left leg irradiating to the dorsal side of the ankle and the foot which lasted over six months. These pains were made worse by effort. Clinical examination showed partial sensitivity deficit and positive Tinel’s sign in the territory of the superficial peroneal nerve (A). Electromyography (EMG) confirmed peroneal nerve involvement with decreased amplitude of sensory potentials. X-ray ruled out the presence of an underlying osteo articular cause (B). Ultrasound objectified well-defined homogeneous tissue formation (C) while MRI showed fusiform mass centered on the nerve with contrast-enhanced T1-weighted hyposignal and T2-weighted hypersignal (D). Conservative surgical resection with sparing of nerve fascicles (E,F) and anatomopathological examination confirmed the diagnosis of benign schwannoma (G). At 1-month follow-up, symptoms had disappeared with sensitivity conservation in the territory of this nerve. The diagnosis of schwannoma is difficult due to the absence of swelling and because schwannoma may mimic entrapment neuropathy, as in the case of our patient. Schwannomas are resectable peripheral nerve tumors. Their prognosis is excellent after adapted surgical treatment with sparing of nerve fascicles.

## Image en médecine

Nous rapportons le cas d'un patient âgé de 61 ans sans antécédents pathologiques particuliers. Il se plaint depuis 6 mois de douleurs avec paresthésies au niveau de la face antérolatérale de la jambe gauche irradiant sur la face dorsale de la cheville et du pied.. Ces douleurs sont exacerbées par l'effort. L'examen clinique a objectivé un déficit partiel de la sensibilité et un signe de Tinel positif dans le territoire du nerf fibulaire superficiel (A). L'EMG a confirmé l'atteinte de ce nerf avec une diminution d'amplitude des potentiels sensitifs. Une radiographie a éliminé une cause ostéo articulaire sous-jacente (B). L'échographie a objectivé une formation tissulaire homogène bien limitée (C) et l'IRM. Une masse fusiforme centrée sur le nerf en hyposignal en T1, et hypersignal en T2 réhaussée par le produit de contraste (D). L'exérèse chirurgicale conservatrice respectant les fascicules nerveux (E, F) et l'anatomopathologie ont confirmé le diagnostic de schwannome (G) et son caractère bénin. A 1 mois de recul, le patient a vu disparaitre sa symptomatologie avec conservation de la sensibilité dans le territoire de ce nerf. En l'absence de tuméfaction, le diagnostic d'un schwannome est difficile et peut mimer un syndrome canalaire comme le cas dans notre observation. Les schwannomes sont des tumeurs nerveuses périphériques extirpables. Leur pronostic reste excellent après un traitement chirurgical adapté respectant les fascicules nerveux.

**Figure 1:**
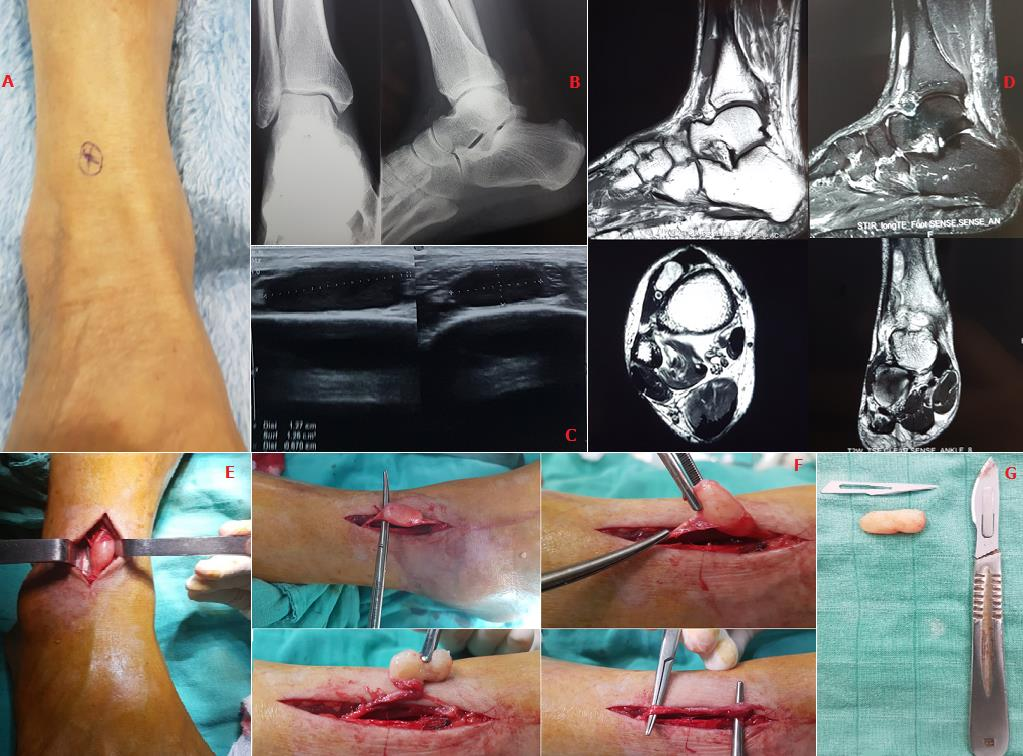
(A) image clinique qui montre la localisation du point douloureux chez notre patient (signe de tinel positif); (B) radiographie standard éliminant une cause ostéoarticulaire sous-jacente; (C) échographie: formation tissulaire oblongue homogène bien limitée; (D) aspect de la tumeur sur l’IRM: masse tissulaire bien limitée, fusifome, en hyposignal en T1, en hypersignal T2 et STIR, se réhaussant faiblement après injection de godolinium; (E) image péropératoire: localisation de la tumeur; (F) images peropératoires: les étapes de dissection de la tumeur autour du nerf (énucléation); (G) aspect final de la pièce tumorale

